# Supplementary material for: A Six-Week Follow-Up Study on the Sustained Effects of Prolonged Water-Only Fasting and Refeeding on Markers of Cardiometabolic Risk
Source: Nutrients. 2022 Oct 15;14(20):4313. doi: 10.3390/nu14204313 (PMC9612103; doi:10.3390/nu14204313)
Supplement: Supplementary file 1 [file nutrients-14-04313-s001.zip › nutrients-1936888-supplementary.pdf]

Supplemental Materials

SOS-Free Diet Screener Supplemental Methods

SOS-Free Diet Screener

Instructions

- Please consider what you have typically eaten over the last **four weeks** when answering the following questions.
- Complete each question as best you can. Estimate if you are not sure. A guess is better than leaving a blank.
- Provided answer selection for each question included: 0, 1 per month, 2-3 per month, 1 per week, 2 per week, 3-4 per week, 5-6 per week, 1 per day, 2 per day, 3 per day, 4 per day, ≥ 5 per day

Questions

FRUITS AND VEGETABLES

1. How often did you eat a serving of **vegetables** (including fresh, frozen, or canned without added salt)?

1 serving = 2 cups of leafy salad greens, 1 cup of raw, cooked, or canned vegetables

2. How often did you eat a serving of **fruit** (including fresh, frozen, or canned without added sugar)?

1 serving = 1 cup, 1 piece of fruit, or ½ cup dried fruit

3. How often did you eat a serving of **avocado** (including guacamole)?

1 serving = ¼ of a whole avocado or ¼ cup of guacamole

GRAINS

4. How often did you eat a serving of ***intact*** **whole grains** (including brown rice, quinoa, millet, barley, buckwheat, wheat berries, etc.)?

1 serving = ½ cup

5. How often did you eat products made with a serving of **whole grain *flour*** (including whole grain pasta, waffles, pancakes, muffins, baked goods, whole grain cereal, etc.)? *Please do not include bean pastas.*

1 serving = ½ cup of whole grain pasta, 1 slice whole grain bread, 1 whole grain waffle, pancake, muffin, or baked good

6. How often did you eat products made with a serving of ***refined* grains** (including white bread, white rice, crackers, ready-to-eat cereal, cereal bars, baked goods, etc.)?

1 serving = 1 slice of white bread; 1 cup ready-to-eat cereal, 1 serving conventional baked good

LEGUMES, NUTS, AND SEEDS

7. How often did you eat a serving of **beans, lentils,** or **split peas** (including black beans, kidney beans, chickpeas, etc.)? *Please include tofu, tempeh, soymilk, and bean pasta.*

1 serving = ½ cup

8. How often did you eat a serving of **nuts**, **seeds, or coconut** (including peanuts, peanut butter, other nut butters, nut milks, etc.)?

1 serving = 1 ounce of nuts or seeds, 2 tablespoons of nut or seed butter, ½ cup shredded coconut, or ¼ cup coconut milk

ANIMAL FOODS

9. How often did you eat a serving of **red meat** (including steak, pork, hamburgers, hot dogs, bacon, etc.)? *Please do not include vegetarian "meats."*

1 serving = 3 ounces of a steak, 1 hamburger, 2 hotdogs, or 7 strips of bacon

10. How often did you eat a serving of **turkey, chicken,** or **other poultry** (including duck, quail, grouse, etc.)?

1 serving = 3 ounces

11. How often did you eat a serving of **fish** or **seafood** (including oysters, clams, sushi, lox, fried fish, etc.)?

1 serving = 3 ounces

12. How often did you eat a serving of **dairy milk, yogurt, butter,** or **cheese** (including cottage cheese, cream cheese, coffee creamer, ice cream, frozen yogurt, etc.)? *Please do not include non-dairy milks and cheeses or the milk or butter in baked goods.*

1 serving = 1 cup of milk or yogurt, 1½ ounce of cheese, 1 tablespoon of butter

13. How often did you eat a serving of **eggs** (including whole eggs or egg whites, eaten in salads, quiche, and souffles)? *Please do not include egg substitutes and do not count eggs in baked goods and desserts.*

1 serving = 1 egg

SOS FOODS AND BEVERAGES

14. How often did you eat **prepared foods containing salt** (such as restaurant food, frozen meals, canned foods with added salt, vegetables or beans, non-dairy cheese, and dishes prepared by friends or family)?

15. How often did you add **salt** (including sea salt, Himalayan salt, table salt, soy sauce, Bragg's liquid aminos, typically canned vegetables, etc.) at the table to foods that you ate at home?

16. How often did you (or someone who cooks for you) add **salt** (including sea salt, Himalayan salt, table salt, soy sauce, Bragg's liquid aminos, typically canned vegetables, etc.) to foods that were cooked at your home?

17. How often did you eat **prepared foods containing oil** (such as including restaurant food, frozen meals, cookies, baked goods, other prepared foods such as vegetable or bean dishes, and non-dairy cheese)? It's okay if some of these are the same foods you were thinking of when answering question 14.

18. How often did you add **oil** (including olive oil, butter, flax oil, coconut oil, vegetable oil, etc.) at the table to foods that you ate at home?

19. How often did you (or someone who cooks for you) add **oil** (including olive oil, butter, flax oil, coconut oil, vegetable oil, etc.) to foods that were cooked at your home?

20. How often did you eat prepared **sweetened foods** (such as candy, chocolate, pastry, and sweetened non-dairy yogurts and desserts, etc.) and **sweetened beverages** (including soda, pop, energy drinks, sweetened coffee, sweetened plant-based milks, cocktails, etc.) prepared with **honey**, **maple syrup**, **agave**, or **sugar**?

21. How often did you add **sugar** (including white sugar, brown sugar, honey, maple syrup, agave, etc.) at the table to foods that you ate at home?

22. How often did you (or someone who cooks for you) add **sugar** (including white sugar, brown sugar, honey, maple syrup, agave, etc.) to foods that were cooked at your home?

SUPPLEMENTS

23. How often did you take **vitamin B12** (including from fortified foods and beverages, multivitamins, or pure B12 supplements)?

24. Which **additional supplements** do you take?

- None
- Omega-3 (including flax oil, fish oil, algae-derived, and others)
- Vitamin D (including D2 or D3)
- Iodine
- Probiotics
- Calcium
- Multi-vitamin/multi-mineral
- Fiber
- Food-derived supplements
- Other: __________________

ADDITIONAL SUBSTANCES

25. How often did you drink a serving of **alcoholic** **beverages** (such as beer, wine, and spirits)?

1 serving = 12 oz beer, 5 oz wine, or 1.5 oz distilled spirits

26. How often did you drink a serving of **<u>caffeinated</u> beverages** (such as coffee, black tea, and energy drinks)? *Please do not include decaffeinated coffee or tea.*

1 serving = 8 oz coffee, 16 oz black tea, 8 oz energy drink

27. How often do you use **tobacco** products (including cigarettes, e-cigarettes, and chewing tobacco)

1 serving = 1 cigarette or 1 packet of chewing tobacco

SOS-Free Diet Screener Scoring

The following equation was used to calculate the non-adherence score for the SOS-Free Diet Screener:

Non-adherence score = (5 - vg) + (4 - fr) + (2-ns) + (1-vb) + (en) + (ea) + (ap) + (sa) + (ao) + (su) + (gr) + (as)

Where vg = servings of vegetables; fr = servings of fruit; ns = servings of nuts and seeds; vb = servings of vitamin B12; en = excess servings of nuts and seeds; ea = excess servings of avocado; ap = servings of animal products; sa = servings of added salt; ao = servings of added oil; su = servings of added sugar; gr = servings of refined or glutenous grains; as = servings of additional substances

Consumption of vg, fr, and vb in excess of the recommended number of servings does not affect the total score. Intact whole grains and legumes were not included in the scoring equation as they are not required for adherence to the SOS-Free Diet. To compute the non-adherence score, the components in the equation were mapped onto variables in the raw data file, where “sos_free” means that the variable was obtained from the raw data file and “n” means that the variable has been converted to daily intake units:

- ns = nuts_seeds_sos_free_n

- en
  - If nuts_seeds_sos_free_n – 4 > 0 then use nuts_seeds_sos_free_n – 4
  - If nuts_seeds_sos_free_n – 4 ≤ 0 then use 0
- ea
  - If avocado_sos_free_n – ½ > 0 then use avocado_sos_free_n – ½
  - If avocado_sos_free_n – ½ ≤ 0 then use 0
- vg = vegetables_sos_free_n
- vg_cond
  - If vegetables_sos_free_n ≤ 5 then use 5 – vegetables_sos_free_n
  - If vegetables_sos_free_n > 5 then use 0
- fr = fruit_sos_free_n
- fr_cond
  - If fruit_sos_free_n ≤ 4 then use 4 - fruit_sos_free_n
  - If fruit_sos_free_n > 4 then use 0
- vb = b12_sos_free_n
- vb_cond
  - If b12_sos_free_n ≤ 1 then use 1 – b12_sos_free_n
  - If b12_sos_free_n > 1 then use 0
- ap = red_meat_sos_free_n + turkey_chicken_sos_free_n + fish_seafood_sos_free_n + dairy_sos_free_n + eggs_sos_free_n
- whole_grain_flour_cond
  - If grain_flour_sos_free_n - 1/3 > 0 then use grain_flour_sos_free_n - 1/3
  - If grain_flour_sos_free_n - 1/3 ≤ 0 then use 0

- gr = + whole_grain_flour_cond + refined_grains_sos_free_n
- as = alcohol_sos_free_n + caffeinated_sos_free_n + tobacco_sos_free_n
- su = added_sugar_ n
- ao = added_oil_n
- sa = added_salt_sos_free_n

Using this map, the non-adherence score from above was reformatted as follows:

Non-adherence score = vg_cond + fr_cond + (2-ns) + vb_cond + en + ea + ap + sa + ao + su + gr + as

Table S1: SOS- Free Diet Screener Serving Size Individual Survey Responses

Number of servings	0 per month	1 per month	2-3 per month	1 per week	2 per week	3-4 per week	5-6 per week	1 per day	2 per day	3 per day	4 per day	≥ 5 per day
**Vegetables**												
**BL**	NA	NA	NA	1 (2.6%)	0 (0%)	7 (18%)	1 (2.6%)	4 (11%)	5 (13%)	11 (29%)	1 (2.6%)	8 (21%)
**FU**	NA	NA	NA	0 (0%)	0 (0%)	1 (3.0%)	0 (0%)	1 (3.0%)	7 (21%)	5 (15%)	7 (21%)	12 (36%)
**Fruit**												
**BL**	NA	2 (5.3%)	2 (5.3%)	2 (5.3%)	1 (2.6%)	5 (13%)	1 (2.6%)	6 (16%)	8 (21%)	5 (13%)	3 (7.9%)	3 (7.9%)
**FU**	NA	0 (0%)	0 (0%)	0 (0%)	2 (6.1%)	0 (0%)	1 (3.0%)	7 (21%)	8 (24%)	8 (24%)	2 (6.1%)	5 (15%)
**Avocado**												
**BL**	4 (11%)	3 (7.9%)	9 (24%)	3 (7.9%)	3 (7.9%)	8 (21%)	3 (7.9%)	3 (7.9%)	1 (2.6%)	0 (0%)	1 (2.6%)	NA
**FU**	5 (15%)	2 (6.1%)	3 (9.1%)	4 (12%)	6 (18%)	6 (18%)	0 (0%)	6 (18%)	0 (0%)	1 (3.0%)	0 (0%)	NA
**Whole grain**												
**BL**	2 (5.3%)	3 (7.9%)	4 (11%)	4 (11%)	2 (5.3%)	8 (21%)	6 (16%)	4 (11%)	5 (13%)	0 (0%)	NA	NA
**FU**	0 (0%)	0 (0%)	1 (3.0%)	1 (3.0%)	4 (12.1%)	6 (18.2%)	2 (6.1%)	8 (24.2%)	8 (24.2%)	3 (9.1%)	NA	NA
**Grain flour**												

**BL**	6 (16%)	3 (7.9%)	4 (11%)	5 (13%)	7 (18%)	6 (16%)	1 (2.6%)	2 (5.3%)	3 (7.9%)	NA	NA	1 (2.6%)
**FU**	6 (18%)	5 (15%)	3 (9.1%)	2 (6.1%)	8 (24%)	5 (15%)	1 (3.0%)	3 (9.1%)	0 (0%)	NA	NA	0 (0%)
**Refined grain**												
**BL**	8 (21%)	3 (7.9%)	5 (13%)	2 (5.3%)	5 (13%)	5 (13%)	4 (11%)	1 (2.6%)	0 (0%)	4 (11%)	NA	1 (2.6%)
**FU**	16 (48%)	4 (12%)	2 (6.1%)	1 (3.0%)	5 (15%)	1 (3.0%)	1 (3.0%)	1 (3.0%)	1 (3.0%)	1 (3.0%)	NA	0 (0%)
**Legumes**												
**BL**	2 (5.3%)	2 (5.3%)	2 (5.3%)	2 (5.3%)	6 (16%)	9 (24%)	4 (11%)	4 (11%)	5 (13%)	2 (5.3%)	0 (0%)	NA
**FU**	0 (0%)	0 (0%)	0 (0%)	0 (0%)	1 (3.0%)	7 (21%)	6 (18%)	7 (21%)	8 (24%)	3 (9.1%)	1 (3.0%)	NA
**Nuts/Seeds**												
**BL**	2 (5.3%)	3 (7.9%)	2 (5.3%)	3 (7.9%)	2 (5.3%)	7 (18%)	3 (7.9%)	8 (21%)	6 (16%)	2 (5.3%)	NA	NA
**FU**	3 (9.1%)	1 (3.0%)	1 (3.0%)	1 (3.0%)	4 (12%)	7 (21%)	3 (9.1%)	7 (21%)	4 (12%)	2 (6.1%)	NA	NA
**Red meat**												
**BL**	20 (53%)	4 (11%)	3 (7.9%)	2 (5.3%)	3 (7.9%)	3 (7.9%)	1 (2.6%)	2 (5.3%)	NA	NA	NA	NA
**FU**	27 (82%)	1 (3.0%)	1 (3.0%)	2 (6.1%)	1 (3.0%)	0 (0%)	0 (0%)	1 (3.0%)	NA	NA	NA	NA
**Turkey/chicken**												
**BL**	19 (50%)	1 (2.6%)	3 (7.9%)	3 (7.9%)	7 (18%)	2 (5.3%)	1 (2.6%)	1 (2.6%)	1 (2.6%)	NA	NA	NA
**FU**	28 (85%)	2 (6.1%)	1 (3.0%)	2 (6.1%)	0 (0%)	0 (0%)	0 (0%)	0 (0%)	0 (0%)	NA	NA	NA
**Fish/Seafood**												
**BL**	15 (39%)	7 (18%)	5 (13%)	2 (5.3%)	6 (16%)	2 (5.3%)	NA	1 (2.6%)	NA	NA	NA	NA
**FU**	26 (79%)	3 (9.1%)	3 (9.1%)	0 (0%)	1 (3.0%)	0 (0%)	NA	0 (0%)	NA	NA	NA	NA
**Dairy**												
**BL**	17 (45%)	1 (2.6%)	5 (13%)	1 (2.6%)	NA	2 (5.3%)	2 (5.3%)	5 (13%)	1 (2.6%)	3 (7.9%)	1 (2.6%)	NA
**FU**	21 (64%)	4 (12%)	5 (15%)	0 (0%)	NA	2 (6.1%)	0 (0%)	0 (0%)	1 (3.0%)	0 (0%)	0 (0%)	NA
**Eggs**												
**BL**	18 (47%)	3 (7.9%)	1 (2.6%)	2 (5.3%)	3 (7.9%)	4 (11%)	2 (5.3%)	4 (11%)	NA	NA	NA	1 (2.6%)
**FU**	28 (85%)	1 (3.0%)	2 (6.1%)	1 (3.0%)	1 (3.0%)	0 (0%)	0 (0%)	0 (0%)	NA	NA	NA	0 (0%)
**Added salt**												
**BL**	8 (21%)	1 (2.6%)	1 (2.6%)	4 (11%)	4 (11%)	4 (11%)	1 (2.6%)	1 (2.6%)	6 (16%)	3 (7.9%)	1 (2.6%)	4 (11%)
**FU**	15 (45%)	2 (6.1%)	6 (18%)	4 (12%)	1 (3.0%)	3 (9.1%)	1 (3.0%)	1 (3.0%)	0 (0%)	0 (0%)	0 (0%)	0 (0%)
**Added oil**												
**BL**	10 (26%)	3 (7.9%)	7 (18%)	1 (2.6%)	2 (5.3%)	3 (7.9%)	4 (11%)	1 (2.6%)	1 (2.6%)	6 (16%)	0 (0%)	NA
**FU**	26 (79%)	2 (6.1%)	3 (9.1%)	0 (0%)	0 (0%)	1 (3.0%)	0 (0%)	0 (0%)	1 (3.0%)	0 (0%)	0 (0%)	NA
**Added sugar**												
**BL**	16 (42%)	4 (11%)	8 (21%)	2 (5.3%)	1 (2.6%)	2 (5.3%)	1 (2.6%)	1 (2.6%)	2 (5.3%)	1 (2.6%)	NA	NA

**FU**	26 (79%)	1 (3.0%)	6 (18%)	0 (0%)	0 (0%)	0 (0%)	0 (0%)	0 (0%)	0 (0%)	0 (0%)	NA	NA
**Vitamin B12**												
**BL**	10 (26%)	2 (5.3%)	1 (2.6%)	4 (11%)	3 (7.9%)	3 (7.9%)	1 (2.6%)	14 (37%)	0 (0%)	NA	NA	NA
**FU**	6 (18%)	0 (0%)	3 (9.1%)	2 (6.1%)	1 (3.0%)	4 (12%)	4 (12%)	12 (36%)	1 (3.0%)	NA	NA	NA
**Alcohol**												
**BL**	18 (47%)	5 (13%)	4 (11%)	4 (11%)	3 (7.9%)	1 (2.6%)	1 (2.6%)	1 (2.6%)	NA	1 (2.6%)	NA	NA
**FU**	24 (73%)	4 (12%)	2 (6.1%)	0 (0%)	1 (3.0%)	0 (0%)	1 (3.0%)	0 (0%)	NA	1 (3.0%)	NA	NA
**Caffeine**												
**BL**	14 (37%)	2 (5.3%)	0 (0%)	2 (5.3%)	2 (5.3%)	0 (0%)	NA	12 (32%)	5 (13%)	1 (2.6%)	NA	NA
**FU**	23 (70%)	2 (6.1%)	3 (9.1%)	1 (3.0%)	2 (6.1%)	1 (3.0%)	NA	1 (3.0%)	0 (0%)	0 (0%)	NA	NA
**Tobacco**												
**BL**	38 (100%)	NA	NA	NA	NA	NA	NA	NA	NA	NA	NA	NA
**FU**	33 (100%)	NA	NA	NA	NA	NA	NA	NA	NA	NA	NA	NA

Numerical values denote the number (percentage) of participants who selected a given serving size at baseline (N=38) and follow up (N=36). BL, baseline; FU, follow up; NA, no answer.

Table S2. SOS-Free Diet Screener Scoring Serving Size Response at BL and FU

	**BL** (N=38)	**FU** (N=33)
	**Median (IQR)**	
**Vegetables conditional (vg_cond)**	2.00 (2.00, 4.00)	1.00 (0.00, 3.00)
**Fruits (fr)**	1.50 (0.50, 3.00)	2.00 (1.00, 3.00)
**Fruits conditional (fr_cond)**	2.50 (1.00, 3.50)	2.00 (1.00, 3.00)
**Nuts and Seeds (ns)**	0.64 (0.18, 1.00)	0.50 (0.29, 1.00)
**Excess of nuts and/or seeds (en)**	0.00 (0.00, 0.00)	0.00 (0.00, 0.00)
**Vitamin B12 (vb)**	0.29 (0.01, 1.00)	0.79 (0.08, 1.00)

**Vitamin B12 conditional (vb_cond)**	0.71 (0.00, 0.99)	0.21 (0.00, 0.92)
**Avocado (avocado_sos_free_n)**	0.21 (0.08, 0.50)	0.29 (0.08, 0.50)
**Excess avocado (ea)**	0.00 (0.00, 0.00)	0.00 (0.00, 0.00)
**Red meat (red_meat_sos_free_n)**	0.00 (0.00, 0.14)	0.00 (0.00, 0.00)
**Turkey and/or chicken (turkey_chicken_sos_free_n)**	0.02 (0.00, 0.29)	0.00 (0.00, 0.00)
**Fish and/or seafood (fish_seafood_sos_free_n)**	0.03 (0.00, 0.14)	0.00 (0.00, 0.00)
**Dairy (dairy_sos_free_n)**	0.08 (0.00, 0.95)	0.00 (0.00, 0.03)
**Eggs (eggs_sos_free_n)**	0.03 (0.00, 0.50)	0.00 (0.00, 0.00)
**Animal products (ap)**	0.31 (0.03, 2.28)	0.00 (0.00, 0.11)
**Added Salt (sa)**	0.50 (0.10, 2.00)	0.03 (0.00, 0.14)
**Added oil (ao)**	0.08 (0.01, 0.79)	0.00 (0.00, 0.00)
**Added sugar (su)**	0.03 (0.00, 0.13)	0.00 (0.00, 0.00)
**Whole grain flour (grain_flour_sos_free_n)**	0.29 (0.08, 0.50)	0.29 (0.03, 0.50)
**Whole grains conditional (whole_grain_flour_cond)**	0.00 (0.00, 0.17)	0.00 (0.00, 0.17)
**Refined grains (refined_grains_sos_free_n)**	0.29 (0.03, 0.71)	0.03 (0.00, 0.29)
**Total conditional grains (gr)**	0.29 (0.08, 0.95)	0.03 (0.00, 0.45)

**Alcohol (alcohol_sos_free_n)**	0.03 (0.00, 0.14)	0.00 (0.00, 0.03)
**Caffeinated beverages (caffeinated_sos_free_n)**	0.29 (0.00, 1.00)	0.00 (0.00, 0.03)
**Tobacco (tobacco_sos_free_n)**	0.00 (0.00, 0.00)	0.00 (0.00, 0.00)
**Additional substances (as)**	0.75 (0.00, 1.29)	0.00 (0.00, 0.11)
**Total Non-adherence Score**	10 (7, 16)	6 (3, 8)

Median (IQR) serving size of each food group and the total non-adherence score. BL, baseline; FU, follow up; IQR, interquartile range.

Supplemental Figure Legends

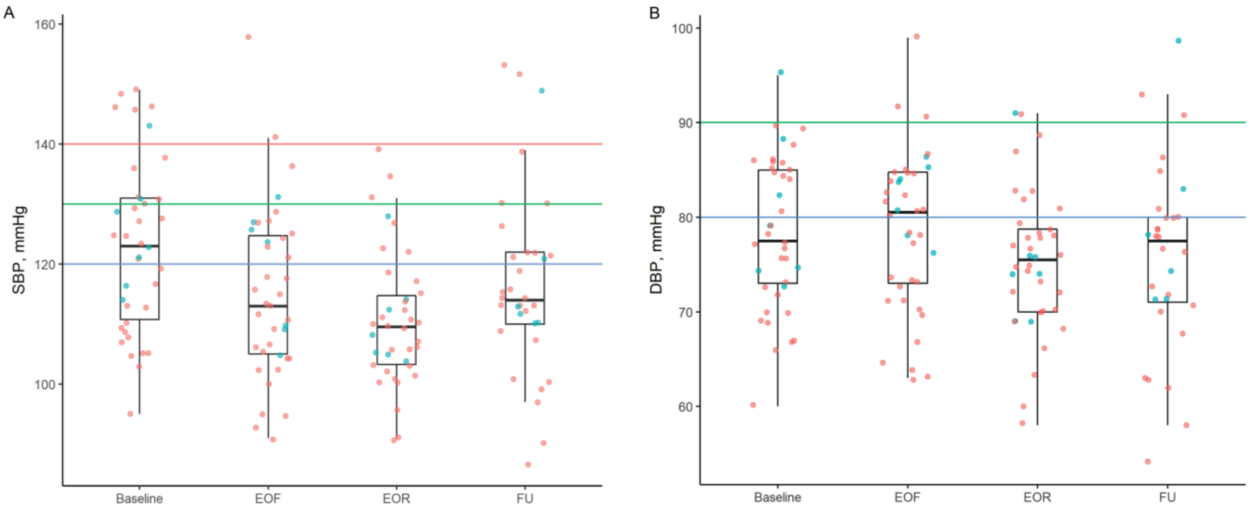

Figure S1. Boxplots of Resting Blood Pressure at Baseline, EOF, EOR, and FU. Box-plot distribution of (A) SBP (mmHg) where SBP below 120 mmHg (blue line) is considered normotensive, SBP between 130 mmHg (green line) and 139 mmHg is considered stage 1 hypertension, and SBP above 140 mmHg (red line) is considered stage 2 hypertension and (B) DBP (mmHg) where DBP below 80 mmHg (blue line) is considered normotensive, DBP between 80 mmHg (blue line) and 89 mmHg is considered stage 1 hypertension, and DBP above 90 mmHg (green line) is considered stage 2 hypertension. Boxplots include the minimum value, first (lower) and third (upper) quartiles, the median, and the maximum value. On all plots, blue dots represent male participants, and pink dots represent female participants. EOF, end of fast; EOR, end of refeed; FU, follow up; SBP, systolic blood pressure; DBP, diastolic blood pressure; mmHg, millimeters of mercury

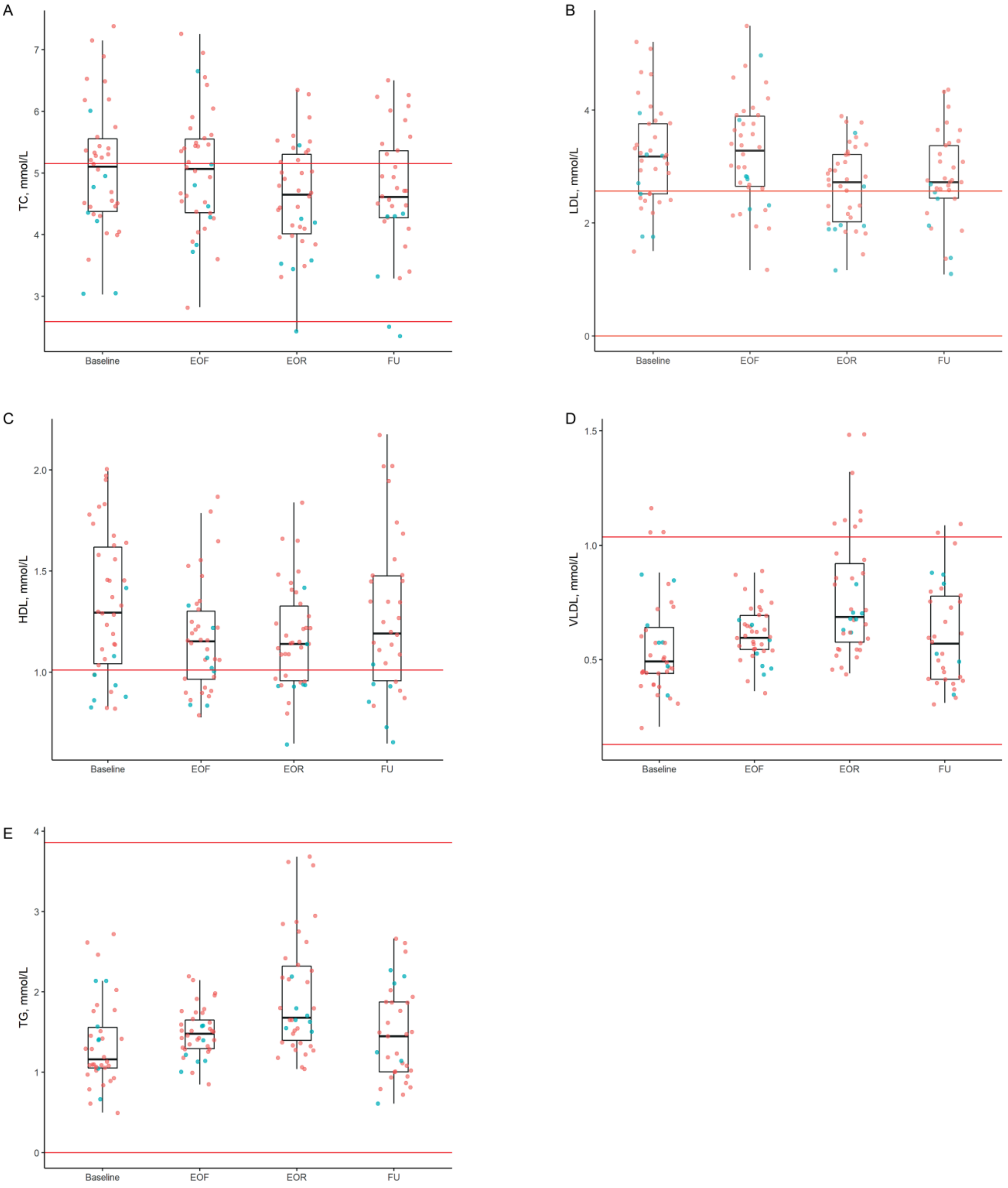

Figure S2. Boxplot of Blood Lipids at Baseline, EOF, EOR, and FU. Box-plot distribution of (A) TC (mmol/L) with values between 2.59 and 5.15 mmol/L (red lines) as the lower and upper limits of the normal reference interval, (B) LDL (mmol/L) with values between 0 and 2.56 mmol/L (red lines) as the lower and upper limits of the normal reference interval, (C) HDL

(mmol/L) with 1 mmol/L (red line) as the lower limit of the normal reference interval, (D) VLDL (mmol/L) with values between 0.13 and 1.04 mmol/L (red lines) as the lower and upper limits of the normal reference interval, and (E) TG (mmol/L) with values between 0 and 3.86 mmol/L (red lines) as the lower and upper limits of the normal reference interval. Boxplots include the minimum value, first (lower) and third (upper) quartiles, the median, and the maximum value. On all plots, blue dots represent male participants, and pink dots represent female participants. EOF, end of fast; EOR, end of refeed; FU, follow up; TC, total cholesterol; LDL, low-density lipoprotein; HDL, high-density lipoprotein; VLDL, very-low-density lipoprotein; TG, triglyceride; mmol, millimole, L, liter.

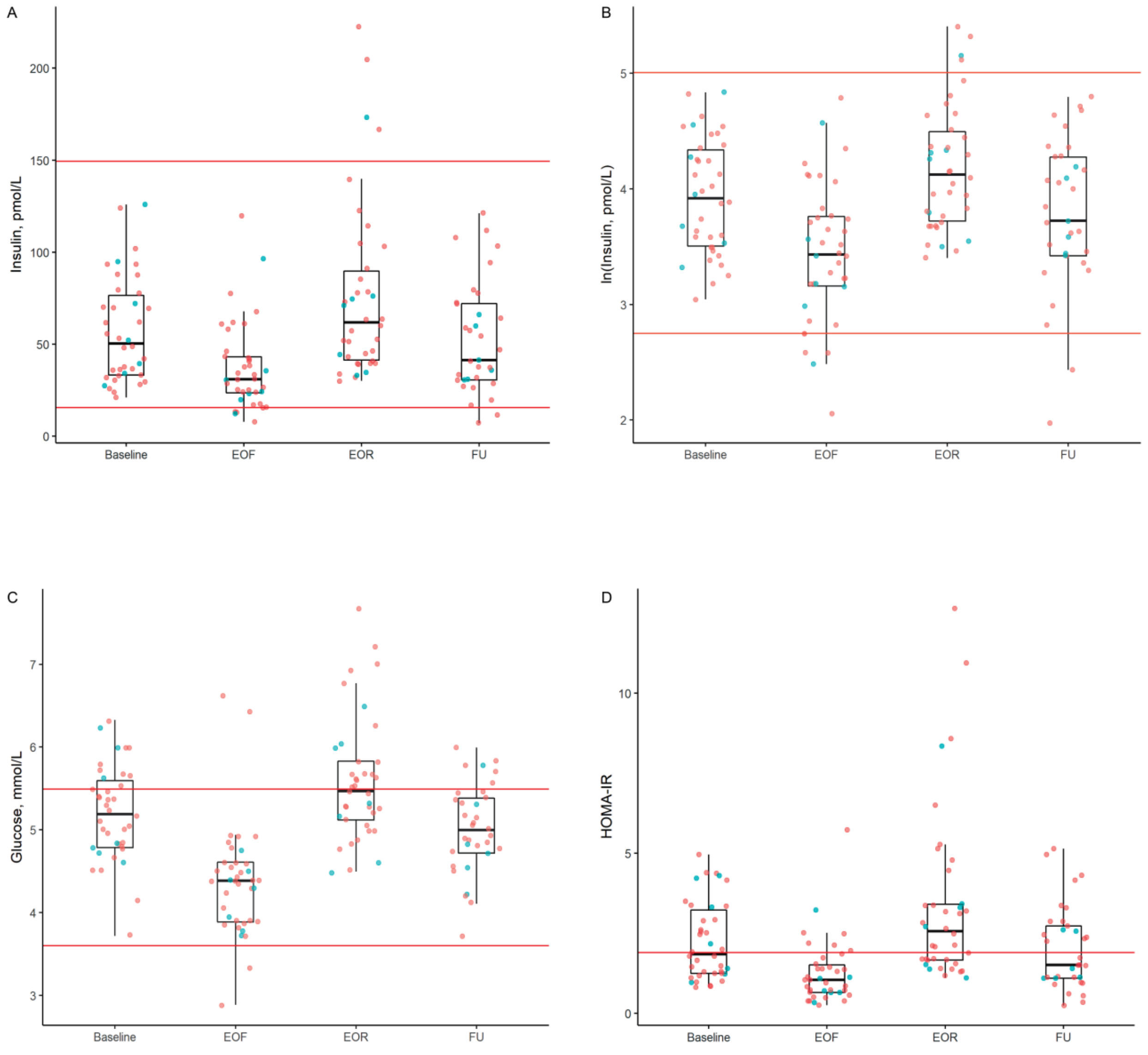

Figure S3. Boxplots of Glycemic Panel at Baseline, EOF, EOR, and FU. Box-plot distribution of (A) Insulin (pmol/L) with values between 15.6 and149.4 pmol/L (red lines) as the lower and upper limits of the normal reference interval, (B) ln(Insulin, pmol/L) with values between 2.75 and 5.01 mmol/L (red lines) as the lower and upper limits of the normal reference

interval; ln, values and reference values are natural log transformed, (C) Glucose (mmol/L) with values between 3.61 and 5.49 mmol/L (red lines) as the lower and upper limits of the normal reference interval, and (D) HOMA-IR; with 1.9 (red line) as the limit of normal reference interval. Boxplots include the minimum value, first (lower) and third (upper) quartiles, the median, and the maximum value. On all plots, blue dots represent male participants, and pink dots represent female participants. EOF, end of fast; EOR, end of refeed; FU, follow up; HOMA-IR, homeostatic model assessment for insulin resistance; mmol, millimole per liter; pmol/L, picomole per liter.
